# Optimizing Antimicrobial Dosing for Critically Ill Patients with MRSA Infections: A New Paradigm for Improving Efficacy during Continuous Renal Replacement Therapy

**DOI:** 10.3390/pharmaceutics14040842

**Published:** 2022-04-11

**Authors:** Jiaojiao Chen, Sihan Li, Quanfang Wang, Chuhui Wang, Yulan Qiu, Luting Yang, Ruiying Han, Qian Du, Lei Chen, Yalin Dong, Taotao Wang

**Affiliations:** 1 Department of Pharmacy, The First Affiliated Hospital of Xi’an Jiaotong University, Xi’an 710061, China; chenjiaojiao123@stu.xjtu.edu.cn (J.C.); lisihan@stu.xjtu.edu.cn (S.L.); xajd14781805941@stu.xjtu.edu.cn (Q.W.); 4121115118@stu.xjtu.edu.cn (C.W.); qiuyulan@stu.xjtu.edu.cn (Y.Q.); 3121115211@stu.xjtu.edu.cn (L.Y.); hry15083352653@stu.xjtu.edu.cn (R.H.); duqian314@stu.xjtu.edu.cn (Q.D.); 2 Department of Hemodialysis, The First Affiliated Hospital of Xi’an Jiaotong University, Xi’an 710061, China; chenlei@mail.xjtu.edu.cn

**Keywords:** continuous renal replacement therapy, critically ill patients, methicillin-resistant *Staphylococcus aureus*, vancomycin, teicoplanin, daptomycin, pharmacokinetics/pharmacodynamics

## Abstract

The dosage regimen of vancomycin, teicoplanin and daptomycin remains controversial for critically ill patients undergoing continuous renal replacement therapy (CRRT). Monte Carlo simulation was applied to identify the optimal regimens of antimicrobial agents in patients with methicillin-resistant *Staphylococcus aureus* (MRSA) infections based on the mechanisms of different CRRT modalities on drug clearance. The optimal vancomycin dosage for patients received a CRRT doses ≤ 30 mL/kg/h was 20 mg/kg loading dose followed by 500 mg every 8 h, while 1 g every 12 h was appropriate when 35 mL/kg/h was prescribed. The optimal teicoplanin dosage under a CRRT dose ≤ 25 mL/kg/h was four loading doses of 10 mg/kg every 12 h followed by 10 mg/kg every 48 h, 8 mg/kg every 24 h and 6 mg/kg every 24 h for continuous veno-venous hemofiltration, continuous veno-venous hemodialysis and continuous veno-venous hemodiafiltration, respectively. When the CRRT dose increased to 30–35 mL/kg/h, the teicoplanin dosage should be increased by 30%. The recommended regimen for daptomycin was 6–8 mg/kg every 24 h under a CRRT dose ≤ 25 mL/kg/h, while 8–10 mg/kg every 24 h was optimal under 30–35 mg/kg/h. The CRRT dose has an impact on probability of target attainment and CRRT modality only influences teicoplanin.

## 1. Introduction

Patients in intensive care units (ICUs) are at risk of Gram-positive infections, especially by methicillin-resistant *Staphylococcus aureus* (MRSA) infections. The MRSA infection rate among ICU patients is higher than 5% [1], which causes a wide range of infections commonly involving skin, soft tissue, bloodstream and pulmonary infections [2]. The emergence of MRSA in ICU is associated with high mortality and worse clinical outcomes [3], which increases the likelihood of mortality by 50% over methicillin-susceptible *Staphylococcus aureus* infections [4]. For MRSA infections, vancomycin and daptomycin were recommended for treatment by the Infectious Diseases Society of America guidelines [5], and teicoplanin was approved for treatment by the British Society for Antimicrobial Chemotherapy [6].

Renal replacement therapy (RRT) is a treatment method that uses blood purification technology to remove solutes to replace impaired renal function. Clinically, RRT with a treatment duration of 24 h or nearly 24 h a day is generally referred to as continuous renal replacement therapy (CRRT) [7]. CRRT is an important life-support technique that is increasingly being applied to critically ill patients [8]. Approximately 10% of ICU patients required CRRT [9], with mortality rates of 30 to 70% [8]. CRRT can relieve the burden of kidneys by continuously removing toxins and correcting the patient’s volume overload. According to the clearing mechanism of convection and diffusion, CRRT can be divided into different modalities including continuous venous-venous hemofiltration (CVVH), continuous venous-venous hemodialysis (CVVHD) and continuous venous-venous hemodiafiltration (CVVHDF). According to the different dilution methods, CVVH and CVVHDF include pre-dilution and post-dilution modalities [8]. Theoretically, drugs with a molecular weight <5000, low plasma protein binding and a small volume of distribution (*V*_d_) can be removed effectively by CRRT [10]. Research has indicated that the vancomycin was reduced by approximately one-fifth during the 12 h CVVH [11]. Teicoplanin and daptomycin are all excreted by the kidney and have a medium molecule size, and therefore might be cleared by CRRT [12,13,14]. 

The pharmacokinetics (PK) of antibacterial agents change significantly during CRRT. An appropriate dosage regimen of antibacterial agents is critically important for critically ill patients. The dosage recommendations of vancomycin, teicoplanin and daptomycin for critically ill patients who undergoing CRRT have been described. However, the dosages are a matter of debate due to small sample sizes and significant heterogeneity in CRRT modality and CRRT dose (the body weight-based effluent flow rate is usually called the CRRT dose) [12,15,16,17,18,19,20,21]. The optimal dosage regimen during CRRT for critically ill patients with MRSA infections is currently unclear and controversial.

Monte Carlo simulation (MCS) is a method which can ‘expand’ the sample size of a study to provide predictions of the likely result of different therapeutic approaches [22]. MCS allows researchers to explore different dosage regimens and targets in virtual clinical trials. It can be valuable for optimizing dosing for patients undergoing CRRT in the absence of large-scale clinical trials.

Therefore, this study aimed to use MCS to describe the effect of different CRRT modalities and CRRT doses on vancomycin, teicoplanin and daptomycin clearance, and determine the optimal dosage of the above antimicrobial agents from a pharmacokinetics/pharmacodynamics (PK/PD) perspective. 

## 2. Materials and Methods

### 2.1. Parameters Collection

A PubMed search was conducted to collect the parameters for vancomycin, teicoplanin and daptomycin for patients undergoing CRRT. Including the PK parameters [*V*_d_ and non-renal clearance (CL_NR_)], the patient demographic data (body weight) and the CRRT settings [sieving coefficient (SC), saturation coefficient (SA), replacement fluid flow rate, dialysate flow rate, CRRT dose, CRRT dose delivered and blood flow rate]. The *V*_d_, CL_NR_, SC, and SA values were collected from PK studies of critically ill patients undergoing CRRT [12,14,19,20,23,24,25,26,27]. The weight and CRRT setting were derived from a large multicenter CRRT trial which included 561 patients [28]. All data were expressed as mean ± standard deviation and range and listed in Table 1.

#### Clearance Calculation

Total body clearance (CL_Total_) was evaluated as the sum of endogenous and extracorporeal clearance (CL_CRRT_). Endogenous clearance included CL_NR_ and residual renal clearance (CL_R_). CL_Total_ was calculated as follows [29]:

CL_Total_ = CL_NR_ + CL_R_ + CL_CRRT_

Most patients who undergoing CRRT were accompanied with severe renal impairment, the CL_R_ of patients applied to the calculation was therefore assumed to be 0 mL/min. In pre-dilution modality, the plasma entering the hemofilter is diluted by replacement fluid, so drug clearance will be lowered than post-dilution modality. The CL_CRRT_ was calculated using the following equations, as modified from a previously reported equation [29]:

1. CVVH modality clearance:
  CL_pre-filter_ = Q_uf_ × SC × Q_plasma_/(Q_plasma_ + Q_uf_)
  CL_post-filter_ = Q_uf_ × SC
2. CVVHD modality clearance:
  CL_CVVHD_ = Q_d_ × SA
3. CVVHDF modality clearance:
  CL_pre-filter_ = (Q_f_ × SC + Q_d_ × SA) × Q_plasma_/(Q_plasma_ + Q_f_)
  CL_post-filter_ = Q_f_ × SC + Q_d_ × SA
  where CL_pre-filter_ and CL_post-filter_ represents the transmembrane clearance during prefilter hemofiltration and prefilter hemofiltration, respectively. SC represents the sieving coefficient which was defined as the ratio of drug concentration in the ultrafiltrate to plasma, SA represents the saturation coefficient which was used to describe the ability of a drug to diffuse through the filter membrane, Q_uf_ represents the ultrafiltration flow rate, Q_plasma_ represents the plasma flow rate, Q_f_ represents the replacement fluid rate used in CVVH, and Q_d_ represents the dialysate fluid rate used in CVVHD.
  Q_plasma_ = Q_b_ × (1 − hematocrit)
  where Q_plasma_ represents the plasma flow rate and Q_b_ represents the blood flow rate.

In the calculation for Q_plasma_, the hematocrit level was assumed to be 30%, as this is a typical level in patients undergoing CRRT [30]. 

### 2.2. PD Data

The MRSA minimum inhibitory concentration (MIC) distributions for three antibacterial agents were obtained from the European Committee on Antimicrobial Susceptibility Testing. (https://mic.eucast.org/, accessed on 24 March 2021) The specific details are shown in Supplementary Materials Table S1.

### 2.3. Monte Carlo Simulation (MCS)

The appropriate trough concentration (*C*_min_) was used as the target for optimizing the loading dose, and area under the concentration time curve over 24 h in steady-state divided by the MIC (AUC_0–24_/MIC) was used for optimizing the maintenance dose. For vancomycin, *C*_min_ ≥ 15 mg/L and AUC_0–24_/MIC ≥ 400 are currently widely accepted to be used for MRSA infections [31]. For teicoplanin, *C*_min_ ≥ 15 mg/L [31] and AUC_0–24_/MIC ≥ 345 [32] are most commonly used. For daptomycin, the targets are *C*_min_ ≥ 3.2 mg/L [33] and AUC_0–24_/MIC ≥ 1061 [34]. 

The *C*_min_ and AUC_0–24_/MIC were calculated by the following equations:

$${\left(C_{n}\right)}_{\min}=\frac{k_{0}}{kV}(e^{\mathrm{kT}}-1)(\frac{1-e^{-\mathrm{nk}\tau }}{1-e^{-k\tau }})\cdot e^{-k\tau }$$

where (*C*_n_)_min_ is the trough concentration after the n-th dose, T is the continuous infusion time, τ is the dosage interval, k is the elimination rate constant (calculated by CL/*V*_d_), and k_0_ is the intravenous drip rate. The formula was used for the calculation of trough concentration under multi-dose intravenous administration.

$${\mathrm{AUC}}_{0–24}/\mathrm{MIC}=\frac{\mathrm{Dose}}{\mathrm{CL}}$$

where Dose is the daily dose, CL is the total drug clearance. The formula was used for the calculation of AUC_0–24_/MIC at steady state under multi-dose intravenous administration.

In this study, MCS was performed for 10,000 replicates using Crystal Ball software (Oracle Corporation, version 11.1.2.4, Redwood Shores, CA, USA). The parameters of body weight, Q_uf_, Q_Plama_, replacement fluid flow rate, dialysate flow rate and CRRT% delivered were assumed to have a standard normal distribution, and *V*_d_, CL_NR_, SC, and SA were assumed to have a log-normal distribution. In addition, to quantify the effect of different CRRT doses on dosage regimens, the fixed CRRT doses of 25 mL/kg/h, 30 mL/kg/h, and 35 mL/kg/h were used in the simulations. The MCS results were expressed as the probability of target attainment (PTA, defined as the probability that at least a specific value of a pharmacodynamic index is achieved at a certain MIC) and cumulative fraction of response (CFR, defined as the expected population probability of target attainment for a specific drug dose and a specific population of microorganisms) [35]. A PTA or CFR value ≥ 90% were considered the minimum of optimal therapy [36].

## 3. Results

### 3.1. Antimicrobial Agent Loading Dose

**Figure 1:**
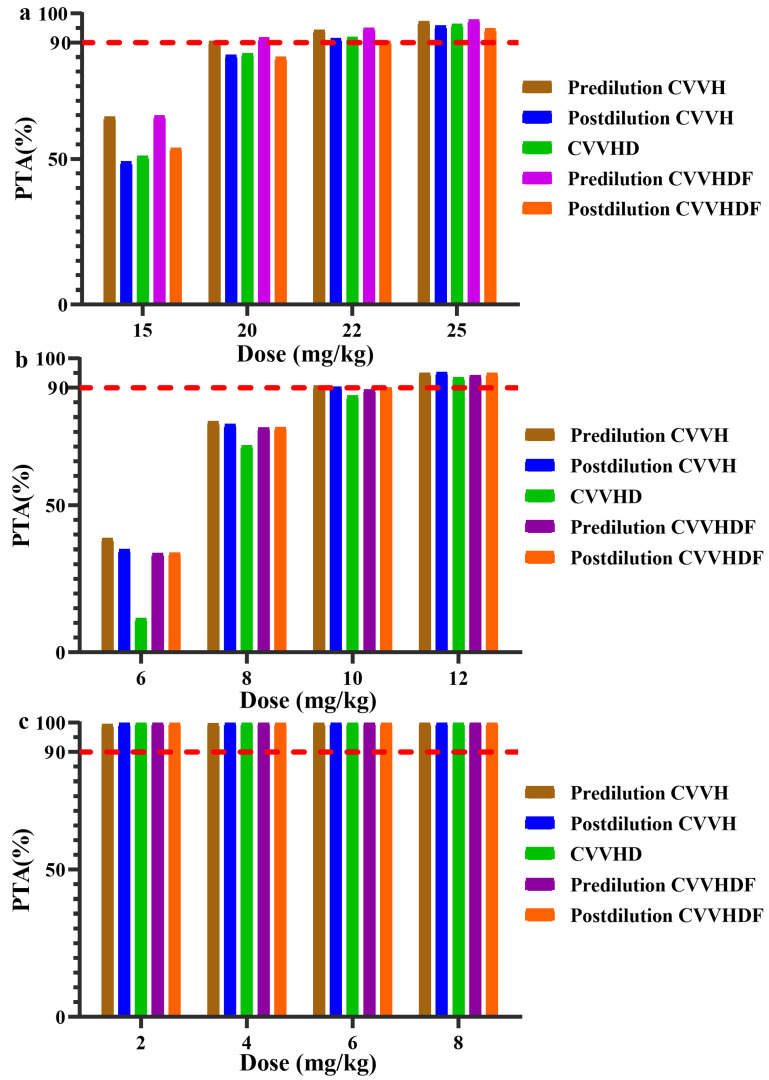
The probability of target attainment (PTA) values of antimicrobial agents in loading doses for patients undergoing different continuous renal replacement therapy modalities. The red dashed horizontal line indicates 90% PTA. (**a**)—Vancomycin; (**b**)—Teicoplanin; (**c**)—Daptomycin.

The PTA values of different loading doses for three antimicrobial agents in different CRRT modalities are presented in Figure 1. A vancomycin loading dose of 20 mg/kg achieved the target (*C*_min_ > 15 mg/L) in five CRRT modalities (Figure 1a). For teicoplanin, four loading doses of 10 mg/kg 12 h achieved the target (*C*_min_ > 15 mg/L) in various CRRT modalities (Figure 1b). For daptomycin, the PTA values of all simulated regimens were 100% (Figure 1c), suggesting that a daptomycin loading dose is not necessary for patients undergoing CRRT.

### 3.2. Antimicrobial Agent Maintenance Dose

**Figure 2:**
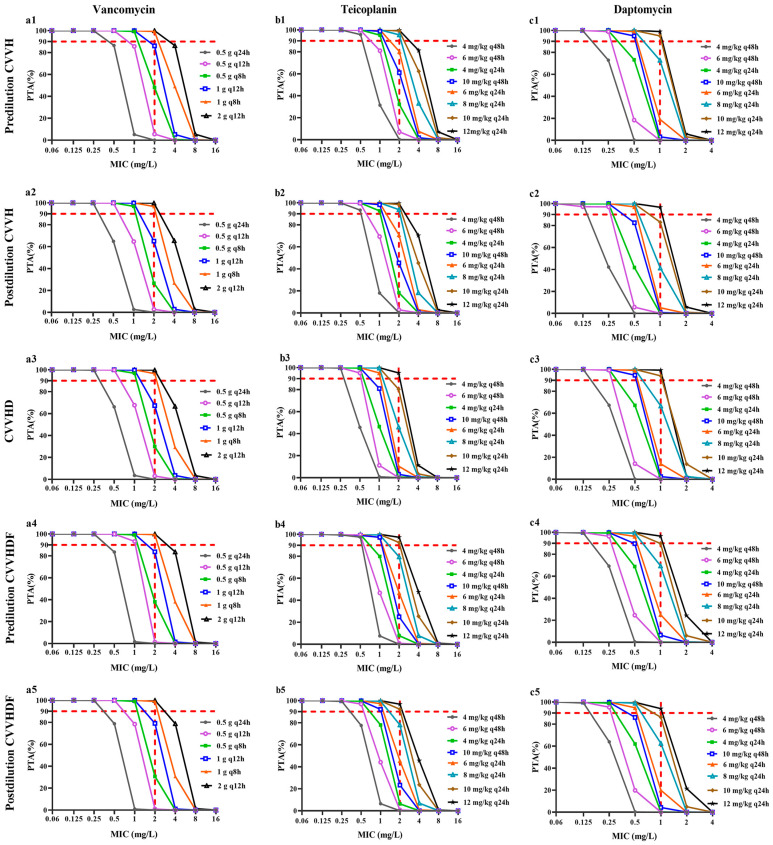
The probability of target attainment (PTA) values of three antimicrobial agents in different maintenance doses for patients undergoing different continuous renal replacement therapy modalities. The red dashed horizontal line indicates 90% PTA. The red dashed vertical line indicates the breakpoints of antimicrobial agents for MRSA. (**a**)—Vancomycin; (**b**)—Teicoplanin; (**c**)—Daptomycin; (**a1**–**c1**)—pre-dilution CVVH; (**a2**–**c2**)—pre-dilution CVVH; (**a3**–**c3**)—CVVHD; (**a4**–**c4**)—pre-dilution CVVHDF; (**a5**–**c5**)—post-dilution CVVHDF.

The PTA of vancomycin regimens according to various MIC and different CRRT modalities are presented in Figure 2(a1–a5). The simulation results indicated that the CRRT modality (CVVH, CVVHD and CVVHDF) had little effect on PTA values. For MIC was 1 mg/L, the PTA values were >90% under the dosage regimen of 0.5 g every 8 h in five CRRT modalities. The regimen of 1 g every 8 h could attain the targets with an MIC of 2 mg/L. The standard dosage regimen of 1 g every 12 h afforded PTA values >90% only for MIC ≤ 1 mg/L.

The PTA results of selected teicoplanin maintenance doses in five CRRT modalities are shown in Figure 2(b1–b5). The simulations indicated that the different CRRT modalities had an effect on teicoplanin PTA values. When MIC was 0.5 mg/L, the dosage regimen of 4 mg/kg every 48 h could achieve the target of AUC_0–24_/MIC ≥ 345 under CVVH and pre-dilution CVVHDF, while it was necessary to increase this dosage to 6 mg/kg every 48 h under CVVHD and post-dilution CVVHDF. The teicoplanin dosage regimens of 4 mg/kg every 24 h and 8 mg/kg every 24 h were acceptable for patients who undergoing CVVH at MIC of 1 mg/L and 2 mg/L, respectively (Figure 2(b1,b2)). However, in the CVVHD modality, the dosage needed adjusting to 6 mg/kg every 24 h and 12 mg/kg every 24 h at MIC of 1 mg/L and 2 mg/L, respectively (Figure 2(b3)). In CVVHDF modality, teicoplanin dosage regimens of 10 mg/kg every 48 h and 10 mg/kg every 24 h afforded PTA values >90% when MIC were 1 and 2 mg/L, respectively (Figure 2(b4,b5)). The standard dosage regimen of 6 mg/kg every 24 h was only appropriate when MIC ≤ 1 mg/L.

Figure 2(c1–c5) shows the PTA values of simulated daptomycin maintenance doses in five CRRT modalities. All of the simulated dosage regimens attained the targets when MIC was 0.125 mg/L regardless of the CRRT modality. When MIC was 0.25 mg/L, 6 mg/kg every 48 h was the optimal dosage for five CRRT modalities. When MIC was 0.5 mg/L and 1 mg/L, the optimized dosage regimens were 10 mg/kg every 48 h and 10 mg/kg every 24 h, respectively, in the pre-dilution CVVH and CVVHD modality (Figure 2(c1,c3)), while in the post-dilution CVVH and CVVHDF modality, the dosage regimen of 6 mg/kg every 24 h was appropriate at MIC of 0.5 mg/L, the dosage needed adjusting to 12 mg/kg every 24 h at MIC of 1 mg/L (Figure 2(c2,c4,c5)).

**Figure 3:**
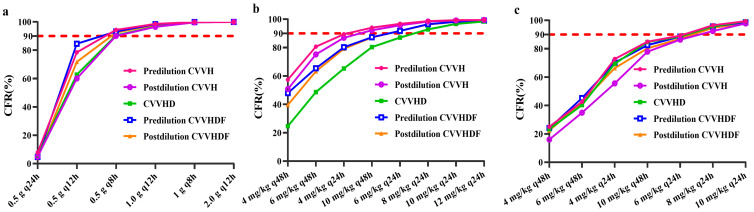
The cumulative fraction of response (CFR) values of three antimicrobial agents in different maintenance doses for patients undergoing continuous renal replacement therapy. The red dashed horizontal line indicates 90% PTA. (**a**)—Vancomycin; (**b**)—Teicoplanin; (**c**)—Daptomycin.

The assessments of CFR values for various dosage regimens of vancomycin, teicoplanin and daptomycin in different CRRT modalities are presented in Figure 3. The vancomycin dosage regimen of 0.5 g every 8 h achieved CFR values >90% regardless of CRRT modality. The acceptable teicoplanin dosage regimens were 10 mg/kg every 48 h in pre-dilution and post-dilution CVVH, 8 mg/kg every 24 h in CVVHD modality, 6 mg/kg every 24 h in pre-dilution and post-dilution CVVHDF, which afforded CFR estimates of 94.01 and 92.16%, 92.81%, 91.73 and 91.73%, respectively. The optimal daptomycin regimen was 6 mg/kg every 24 h regardless of the CRRT modality.

### 3.3. The Impact of CRRT Dose on Maintenance Dose of Antimicrobial Agent

**Figure 4:**
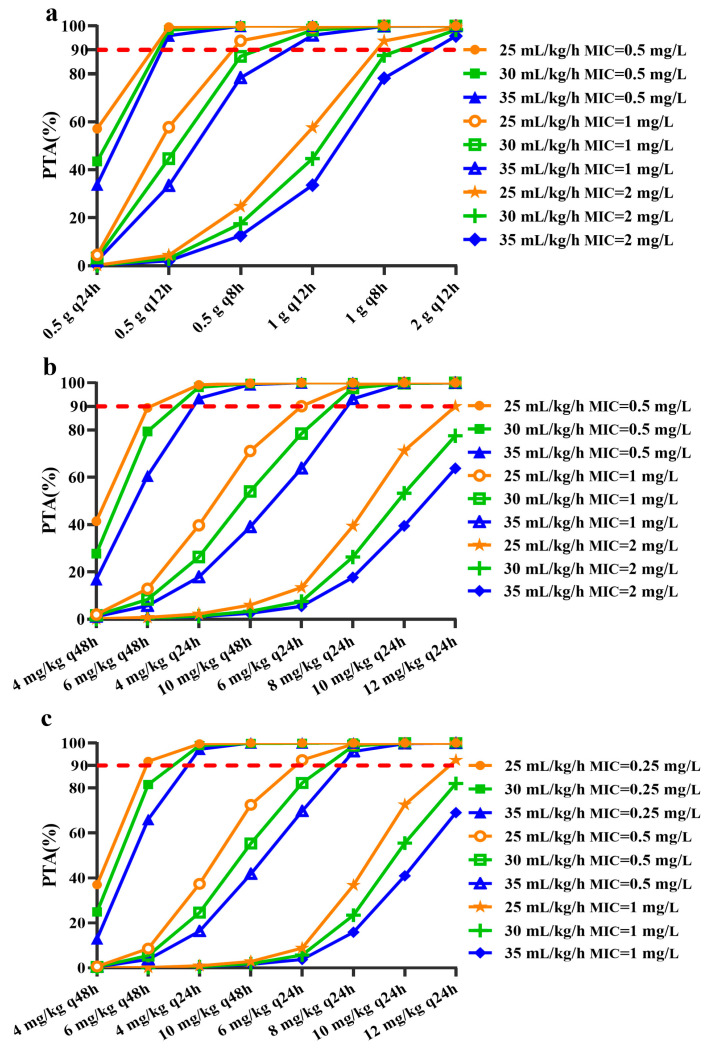
The probability of target attainment (PTA) values in different maintenance doses for patients undergoing different continuous renal replacement therapy (CRRT) doses. CRRT doses were fixed as 25, 30 and 35 mL/kg/h, respectively. The red dashed horizontal line indicates 90% PTA. (**a**)—Vancomycin; (**b**)—Teicoplanin; (**c**)—Daptomycin.

A PTA sensitivity analysis was performed to explore the influence of PK and CRRT parameters on dosage regimens, which indicated that CL_NR_, body weight, and CRRT dose exerted large effect on maintenance dose. Therefore, fixed CRRT doses of 25 mL/kg/h, 30 mL/kg/h and 35 mL/kg/h under the maximum drug clearance CRRT modality (CVVH post-dilution modality for vancomycin and daptomycin, and CVVHD modality for teicoplanin) were used to quantify the impact of CRRT dose on the maintenance doses of three antimicrobial agents (Figure 4). 

The vancomycin dosage regimens under a CRRT dose of 25–30 mL/kg/h were similar to those under the reference CRRT dose (22.0 ± 6.1 mL/kg/h). When a higher CRRT dose of 35 mL/kg/h was prescribed, the vancomycin dosage regimen needed to increase to 1 g every 12 h and 2 g every 12 h at MIC of 1 mg/L and 2 mg/L, respectively (Figure 4a, Table 2).

The teicoplanin recommended dosage regimen was identical under a CRRT dose of 25 mL/kg/h and the reference CRRT dose (22.0 ± 6.1 mL/kg/h). When the CRRT dose increased to 30–35 mL/kg/h, the teicoplanin dosage regimen should be increased appropriately. The dosage regimens of 4 mg/kg every 24 h, 8 mg/kg every 24 h and 12 mg/kg every 24 h produced acceptable PTA for MIC of 0.5 mg/L, 1 mg/L and 2 mg/L, respectively (Table 2).

For the CRRT dose of 25 mL/kg/h, the recommended daptomycin dosage regimen was the same with that under the reference CRRT dose. When the CRRT dose increased to 30–35 mL/kg/h, dosage regimens of 4 and 8 mg/kg every 24 h were appropriate at MIC of 0.25 and 0.5 mg/L, respectively. Meanwhile, a dosage regimen higher than 12 mg/kg every 24 h was recommended when the MIC was 2 mg/L (Table 2).

## 4. Discussion

The current data for quantifying CRRT modality and CRRT dose influence PK is insufficient. The PK altered in critically ill patients undergoing CRRT is extremely complicated, while multiple variables associated with disease status and CRRT settings affect drug clearance [12,15,16,17,18,19,20,21]. These variables make it difficult to generalize dosage regimen recommendations for CRRT patients. This study is a new paradigm to quantify the influence of CRRT dose and modality, and to optimize the dosage regimens for critically ill patients undergoing CRRT.

CRRT patients may have well-preserved CL_NR_ [27], which (16.2 ± 7.0 mL/kg/h) accounted for 30% of the total vancomycin clearance in this study. In addition, 40–50% patients in ICU have hypoalbuminemia [37], which could increase the free drug clearance of CRRT. Additionally, critically ill patients frequently have capillary leakage, fluid therapy, pleural effusion, ascites, and post-surgical indwelling drainage, which may increase *V*_d_ and cause antimicrobial agent dilution in the plasma and extracellular fluids [38]. Given these reasons, the loading dose is crucial for rapidly achieving the PTA target in these patients. 

This study indicated that the vancomycin loading dose regimen of 20 mg/kg followed by 0.5 g every 8 h could achieve the PK/PD target for MRSA under five different CRRT modalities with a CRRT dose <30 mL/kg/h. The recommended vancomycin dosage comparison with other clinical studies are shown in Supplementary Materials Table S2. Our dosage recommendation was consistent with the results of previous studies [11,16,39], which indicated that a 1500 mg loading dose followed by 450–750 mg every 12 h was acceptable for critically ill patients undergoing CRRT. The Japanese Society of Therapeutic Drug Monitoring suggested that a vancomycin loading dose of 15–20 mg/kg in patients undergoing CVVHDF could guarantee the achievement of the target [40].

Moreover, our results suggested that a dosage regimen of 1 g every 8 h could obtain an optimal PTA value when MIC was 2 mg/L. However, the *C*_min_ estimated values of this regimen under five modalities were 41.0, 36.7, 29.8, 33.1, and 32.0 mg/L, which were higher than the therapeutic window for vancomycin (10–20 mg/L). Therefore, vancomycin was not recommended for treating MRSA when the MIC was 2 mg/L. 

There were limited recommended dosage regimens for teicoplanin in patients undergoing CRRT. The simulation results for teicoplanin indicated that the loading dose frequency greatly influenced PTA values. Four loading doses of 10 mg/kg every 12 h or three loading doses of 14 mg/kg every 12 h achieved the PK/PD targets. The PTA values of teicoplanin varied widely among different CRRT modalities in the maintenance doses of 6 mg/kg every 24 h, which afford PTA values of 96.72, 95.65, 87.06, 91.73 and 91.70% in pre-dilution and post-dilution CVVH, CVVHD, pre-dilution and post-dilution CVVHDF modality, respectively, suggesting that the teicoplanin maintenance doses should be adjusted according to the CRRT modality. We therefore recommended four loading doses of 10 mg/kg every 12 h followed by 10 mg/kg every 48 h, 8 mg/kg every 24 h, and 6 mg/kg every 24 h in the CVVH, CVVHD, and CVVHDF modality, respectively. The recommended teicoplanin dosage comparison with other clinical studies are shown in Supplementary Materials Table S2. The loading dose was consistent with an early published PK study in acute renal failure patients who undergoing CVVHD, while the maintenance dose in the present study was much higher than their dosage which was 400 mg every 48–72 h [41], it was presumed that the dialysate flow rate used in the previous study was much lower (16 mL/kg/h) than flow rate (22.0 ± 6.1 mL/kg/h) used in this study. There was no recommended dosage regimen in previous study for patients undergoing CVVH and CVVHDF. The optimal dosage regimen was 10 mg/kg every 48 h and 6 mg/kg every 24 h in the CVVH and CVVHDF modality, respectively, which may provide a reference for clinical practice.

The PTA values of daptomycin were similar among different CRRT modalities, and a dosage regimen of 6–8 mg/kg every 24 h was therefore recommended for the five CRRT modalities. In addition, there was no need to give a loading dose for daptomycin, since the target *C*_min_ could be achieved easily after the first administration. The recommended daptomycin dosage comparison with other clinical studies are shown in Supplementary Materials Table S2. There is currently no recommendation of clinical daptomycin dosage regimen for patients undergoing CVVH. The previous population pharmacokinetic studies [21,24] and review [42] indicated that the dose of 6–8 mg/kg every 24 h may be appropriate during CVVHD and CVVHDF, which was consistent with our study.

When the prescribed CRRT dose was 30 mL/kg/h, vancomycin could still be administered with the dosage regimens under the reference CRRT dose (22.0 ± 6.1 mL/kg/h), while the teicoplanin and daptomycin dosages needed to be increased by 30%. Undoubtedly, if the higher effluent rates of 35 mL/kg/h were utilized, the higher dose (increasing by 30% based on the dosage regimen under the reference CRRT dose) was required to achieve the PK/PD target. Higher antimicrobial doses are needed in higher CRRT doses, our results are consistent with a previous published review [43].

This study also had some limitations. First, the renal clearance applied in the simulation was assumed as 0 mL/min because most patients undergoing CRRT had severe renal impairment. In fact, patients may still have partial residual renal clearance. Second, the PD data used in our simulation was based on EUCAST MIC distributions so we can only consider MIC at a specific value. Indeed, MICs may increase over time. Additionally, although the CRRT settings we selected from the largest number of patients (n = 561) covered most clinical CRRT settings, CRRT practices that differ substantially from in this study will likely produce different results. Finally, due to insufficient data on toxicity threshold, we did not consider the probabilities of toxicity from our dosage regimens. 

## 5. Conclusions

MCS was applied to determine the optimal dosage of vancomycin, teicoplanin and daptomycin based on the mechanisms of different CRRT modalities and CRRT dose on drug clearance. Antimicrobial dosing is should be optimized in different CRRT doses, and CRRT modality only influences teicoplanin dosage. This study provides theoretical evidence for the reasonable application of antimicrobial agents for critically ill patients undergoing CRRT. Clinical studies are needed to validate this recommendation.

## Figures and Tables

**Table 1:** Demographic and pharmacokinetic variables.

Antimicrobial Agents	Vancomycin [17,18]	Teicoplanin [12,13,19,21]	Daptomycin [22,23,24]
*V*_d_	0.57 ± 0.26 L/kg (0.17–1.37)	1.60 ± 0.70 L/kg (1.10–2.10)	6.33 ± 1.65 L (5.67–7.00)
CL_NR_ (mL/min)	16.2 ± 7.0 (3.8–23.3)	6.3 ± 2.2 (0–10.6)	5.0
SC	0.73 ± 0.10 (0.43–0.89)	0.14 ± 0.03 (0–1.0)	0.19 ± 0.02 (0–1.0)
SA	0.71 ± 0.14 (0–1.0)	0.33 ± 0.02 (0–1.0)	0.15 ± 0.01 (0–1.0)
Weight (kg)	84.1 ± 18.9		
CRRT% delivered	95.0 ± 35.0		
CRRT dose (mL/kg/h)	22.0 ± 6.1		
Dialysate fluid rate (mL/h)	820.0 ± 250.0		
Replacement fluid rate (mL/h)	830.0 ± 249.0		
Blood flow (mL/min)	140.0 ± 40.0		

*V*_d_—volume of distribution; CL_NR_—non-renal clearance; SC—sieving coefficient; SA—saturation coefficient. Data were expressed as mean ± standard deviation (range).

**Table 2:** The dosage recommendations of three antimicrobial agents for treating MRSA infections with various MICs for critically ill patients undergoing CRRT.

Antimicrobial Agents	CRRT Modalities	CRRT Doses (mL/kg/h)	MIC (mg/L)			
			0.25	0.5	1	2
Vancomycin	CVVH ^a^	25–30	-	0.5 g q12 h	0.5 g q8 h	1 g q 8 h
	CVVH ^a^	35	-	0.5 g q12 h	1 g q12 h	2 g q12 h
Teicoplanin	CVVH	25	-	4 mg/kg q48 h	4 mg/kg q24 h	8 mg/kg q24 h
	CVVHD	25	-	6 mg/kg q48 h	6 mg/kg q24 h	12 mg/kg q24 h
	CVVHDF	25	-	6 mg/kg q48 h	10 mg/kg q48 h	10 mg/kg q24 h
	CVVH	30–35	-	6 mg/kg q48 h	6 mg/kg q24 h	10 mg/kg q24 h
	CVVHD	30–35	-	4 mg/kg q24 h	8 mg/kg q24 h	>12 mg/kg q24 h
	CVVHDF	30–35	-	4 mg/kg q24 h	6 mg/kg q24 h	12 mg/kg q24 h
Daptomycin	CVVH ^a^	25	6 mg/kg q48 h	6 mg/kg q24 h	12 mg/kg q24 h	-
	CVVH ^a^	30–35	4 mg/kg q24 h	8 mg/kg q24 h	>12 mg/kg q24 h	-

CRRT—continuous renal replacement therapy; CVVH—continuous venous-venous hemofiltration; CVVHD—continuous venous-venous hemodialysis; MIC—minimum inhibitory concentration. ^a^ The dosage regimens in CVVHD and CVVHDF were identical with the dosage regimens in CVVH.

## Data Availability

The datasets used during the current study are available from the corresponding author on reasonable request.

## Supplementary Materials

The following supporting information can be downloaded at: https://www.mdpi.com/article/10.3390/pharmaceutics14040842/s1, Table S1: The frequency distribution of three antimicrobial agents MICs for MRSA; Table S2: Comparison of dosage recommendation of three antimicrobial agents with other clinical studies. References [44,45,46,47,48] are cited in the Supplementary Materials.
