# Supplementary material for: Optimizing Antimicrobial Dosing for Critically Ill Patients with MRSA Infections: A New Paradigm for Improving Efficacy during Continuous Renal Replacement Therapy
Source: Pharmaceutics. 2022 Apr 11;14(4):842. doi: 10.3390/pharmaceutics14040842 (PMC9031498; doi:10.3390/pharmaceutics14040842)
Supplement: Supplementary file 1 [file pharmaceutics-14-00842-s001.zip › pharmaceutics-1622612-supplementary.pdf]

pharmaceutics

MDPI

Optimizing Antimicrobial Dosing for Critically Ill Patients with MRSA infections: A New Paradigm for Improving Efficacy during Continuous Renal Replacement Therapy

Jiaojiao Chen, Sihan Li, Quanfang Wang, Chuhui Wang, Yulan Qiu, Luting Yang, Ruiying Han, Qian Du, Lei Chen, Yalin Dong and Taotao Wang

Table S1. The frequency distribution of three antimicrobial agents MICs for MRSA.

**Antimicrobial agents**	*n*	**MIC (mg/L)**										**Susceptibility breakpoint (mg/L)**
		**0.03**	**0.06**	**0.125**	**0.25**	**0.5**	**1**	**2**	**4**	**8**	**16**	
Vancomycin	2574					4.00	86.32	9.63	0.04			2 [a]
Teicoplanin	566	0.18	0.00	0.53	5.48	39.58	42.23	10.78	1.06	0.00	0.18	2 [a]
Daptomycin	448			3.57	29.24	54.16	12.72					1 [a]

MIC: minimum inhibitory concentration; EUCAST: European Committee on Antimicrobial; MRSA: Methicillin resistant *Staphylococcus aureus*.
a. The Susceptibility breakpoint came from EUCAST.

Table S2. Comparison of dosage recommendation of three antimicrobial agents with other clinical studies.

Antimicrobial agents	Reference	Year	Population (Number)	CRRT modalities	CRRT doses	CL $_{Total}$	CL$_{CRRT}$	Dosage recommendation by authors	Dosage recommendation in our study
Vancomycin	Wang et al. [44]	2021	Critically ill patients (11)	CVVH	20–35 mL/kg/h	-	-	10 mg/kg q24h	20 mg/kg loading dose followed by 500 mg q8 h
	Wang et al. [44]	2021	Critically ill patients (11)	CVVH	35–40 mL/kg/h	-	-	5 mg/kg q8h	20 mg/kg loading dose followed by 1000 mg q12 h
	Li et al. [45]	2020	Severe pneumonia patients (10)	CVVH	30–40 mL/kg/h	2.36 ± 0.72 L/h	1.35 ± 0.03 L/h	400–650 mg q12 h	20 mg/kg loading dose followed by 1000 mg q12 h
	Vijsel et al. [39]	2010	Critically ill patients (24)	CVVHD	1000 mL/h	40 mL/min		Intermittent infusion: 1.25–1.5 g q24h Or continuous infusion: 1.5g loading dose followed by of 1–1.5 g IV over 24 h	20 mg/kg loading dose followed by 500 mg q8 h
	Chaijamorn et al. [11]	2011	Acute kidney injury patients (7)	CVVH	800–1200 mL/h	1.59 ± 0.47 L/h	12.11 ± 3.50 mL/min	500–750 mg q12 h	20 mg/kg loading dose followed by 500 mg q8 h
	Seung et al. [20]	2019	Anuria patients	CVVHDF	32.1 ± 7.0 mL/kg/h	11.9 ± 5.4 mL/min	5.9 ± 4.2 mL/min	408.7 ± 197.7 mg q24 h	four loading doses of 10 mg/kg q12 h followed by 8 mg/kg q24 h

Teicoplanin	Bellmann et al. [14]	2010	Most was are sepsis patients (4)	CVVH	35 mL/kg/h	11 ± 4 mL/min	-	1200 mg loading dose followed by 600–1800 mg q24 h	four loading doses of 10 mg/kg q12 h followed by 6 mg/kg q24 h
	Yagasaki et al. [46]	2003	Acute renal failure patients (3)	CVVHDF	-	11.3 ± 9.1 mL/min	3.5 ± 1.1 mL/min	200 mg q48 h or 400 mg q24 h	four loading doses of 10 mg/kg q12 h followed by and 6 mg/kg q24 h
	Wolter et al. [41]	1994	Acute renal failure patients (5)	CVVHD	11.5 mL/kg/h	7.9 mL/min	-	800 mg loading dose followed by 400 mg q 48–72 h	four loading doses of 10 mg/kg q12 h followed by and 8 mg/kg q24 h
Daptomycin	Corti et al. [12]	2013	Anuric patients (9)	CVVHDF (n = 8) CVVHD (n = 1)	36.7± 13 mL/kg/h	10.2 mL/min	4.5 ± 1.5 mL/min	6 mg/kg q 24 h	8 mg/kg q24 h
	Wenisch et al. [47]	2011	Acute renal failure patients (9)	CVVHDF	2000 mL/h	6.1 ± 4.9 mL/min	-	8 mg/kg q 48 h	8 mg/kg q24 h
	Vilay et al. [26]	2011	Sepsis patients (8)	CVVHD	33 ± 5 mL/kg/h	11.3 ± 4.7 mL/min	6.3 ± 2.9 mL/min	8 mg/kg q 48 h	8 mg/kg q24 h
	Khadzhynov et al. [48]	2011	Critically ill patients (8)	CVVHD	30.1 ± 2.7 mL/kg/h	-	40% CL_{Total}	≥ 4 mg/kg q 24 h	8 mg/kg q24 h

CRRT—continuous renal replacement therapy; CVVH—continuous venous-venous hemofiltration; CVVHD—continuous venous-venous hemodialysis.
